# Development of a Novel Clinical Decision Support System for Exercise Prescription Among Patients With Multiple Cardiovascular Disease Risk Factors

**DOI:** 10.1016/j.mayocpiqo.2020.08.005

**Published:** 2020-10-22

**Authors:** Linda S. Pescatello, Yin Wu, Gregory A. Panza, Amanda Zaleski, Margaux Guidry

**Affiliations:** a Department of Kinesiology, University of Connecticut, Storrs, CT; b Department of Preventive Cardiology, Hartford Hospital, Hartford, CT; c Servier Pharmaceuticals, Boston, MA

**Keywords:** ACSM, American College of Sports Medicine, AHA, American Heart Association, AHA_7_CVH, American Heart Association Life’s Simple 7 cardiovascular health scoring system, BG, blood glucose, BMI, body mass index, BP, blood pressure, CVD, cardiovascular disease, CV, cardiovascular, CVH, cardiovascular health, DBP, diastolic blood pressure, Ex R_x_, exercise prescription, FITT, frequency, intensity, time, and type, HDL-C, high-density lipoprotein cholesterol, HTN, hypertension, HR, heart rate, HRR, heart rate reserve, LDL-C, low-density lipoprotein cholesterol, 1-RM, one repetition maximum, P3-EX, prioritize personalize prescribe exercise clinical decision support system, PNF, proprioceptive neuromuscular facilitation, RPE, rating of perceived exertion, SBP, systolic blood pressure, T1DM, type 1 diabetes mellitus, TC, total cholesterol, T2DM, type 2 diabetes mellitus, SOB, shortness of breath, VO_2_R, oxygen uptake reserve, WC, waist circumference

## Abstract

Cardiovascular disease (CVD) risk factors cluster in an individual. Exercise is universally recommended to prevent and treat CVD. Yet, clinicians lack guidance on how to design an exercise prescription (ExR_x_) for patients with multiple CVD risk factors. To address this unmet need, we developed a novel clinical decision support system to prescribe exercise (prioritize personalize prescribe exercise [P3-EX]) for patients with multiple CVD risk factors founded upon the evidenced-based recommendations of the American College of Sports Medicine (ACSM) and American Heart Association. To develop P3-EX, we integrated (1) the ACSM exercise preparticipation health screening recommendations; (2) an adapted American Heart Association Life’s Simple 7 cardiovascular health scoring system; (3) adapted ACSM strategies for designing an ExR_x_ for people with multiple CVD risk factors; and (4) the ACSM frequency, intensity, time, and time principle of ExR_x_. We have tested the clinical utility of P3-EX within a university-based online graduate program in ExR_x_ among students that includes physicians, physical therapists, registered dietitians, exercise physiologists, kinesiologists, fitness industry professionals, and kinesiology educators in higher education. The support system P3-EX has proven to be an easy-to-use, guided, and time-efficient evidence-based approach to ExR_x_ for patients with multiple CVD risk factors that has applicability to other chronic diseases and health conditions. Further evaluation is needed to better establish its feasibility, acceptability, and clinical utility as an ExR_x_ tool.

Cardiovascular disease (CVD) is the leading cause of death and disability in the United States and the world.^1^ The five major CVD risk factors of hypertension, diabetes mellitus, dyslipidemia, obesity, and physical inactivity cluster in an individual. Because of its many health benefits, leading health care experts from all over the world recognize exercise as the most efficient, cost-effective, and accessible “polypill” to prevent and treat more than 35 chronic diseases and health conditions that include CVD and its major risk factors.^2^, ^3^, ^4^, ^5^, ^6^, ^7^, ^8^ Yet, 82% of adults in the United States do not engage in the recommended amounts of exercise to achieve these health benefits.^9^

Unfortunately, despite exercise being recognized as a polypill to improve health, only 30% of primary care physicians recommend exercise to their patients when a physician’s recommendation to exercise is a strong incentive for their patients to exercise.^10^ Physicians and other health care professionals do not recommend exercise because they lack the tools, training, and time to do so.^11^, ^12^, ^13^ Clinical decision support systems have become essential devices for health care providers to streamline information processing, recommend next steps for treatment, and avoid adverse treatment effects.^14^ Yet, to the best of our knowledge, there is no evidenced-based, time-efficient, guided tool for clinicians to prescribe exercise to their patients. This is a critically important unmet need to address given that exercise is universally recommended to prevent, treat, and control CVD along with its major risk factors.^2^, ^3^, ^4^, ^5^, ^6^, ^7^, ^8^

The American College of Sports Medicine (ACSM) exercise preparticipation health screening recommendations no longer include CVD risk factor profiling.^15^^,^^16^ This omission has created confusion in clinicians’ minds of how to design an exercise prescription (ExR_x_) for patients with multiple CVD risk factors.^12^^,^^17^ In parallel, the American Heart Association (AHA) tracks the cardiovascular health (CVH) in the United States with a tool known as Life’s Simple 7 via assessment of physical activity levels and the biomarkers of blood lipids, blood pressure (BP), blood glucose (BG), and body mass index (BMI) that represent the five major CVD risk factors of physical inactivity, dyslipidemia, hypertension (HTN), diabetes mellitus, and obesity, respectively.^18^^,^^19^ Integrating the industry-standard recommendations set forth by the ACSM^15^^,^^16^ and an adapted version of the AHA Life’s Simple 7 cardiovascular health (AHA_7_CVH) scoring system,^18^^,^^19^ we present a novel, evidence-based clinical decision support system (prioritize, personalize, prescribe exercise [P3-EX]) for clinicians to design an ExR_x_ for patients with multiple CVD risk factors who also may have other chronic diseases and health conditions.

## The P3-EX Clinical Decision Support System for Exercise Prescription

The purpose of P3-EX is to provide physicians and other health care professionals with guidance that is evidenced-based and time-efficient on how to design an ExR_x_ for patients with multiple CVD risk factors who may have other chronic diseases and health conditions.^12^^,^^17^ To develop P3-EX, we integrated: (1) the ACSM exercise preparticipation health screening recommendations^15^^,^^16^; (2) an adapted AHA_7_CVH scoring system^18^^,^^19^; (3) adapted ACSM strategies for designing an ExR_x_ for people with multiple CVD risk factors who may have other chronic diseases and health conditions^20^; and (4) the ACSM frequency, intensity, time, and time (FITT) principle of ExR_x_.^20^ We now introduce P3-EX for designing a FITT ExR_x_ for patients with multiple CVD risk factors who may have other chronic diseases or health conditions.

### Step 1. Complete the ACSM Exercise Preparticipation Health Screening

**Figure 1:**
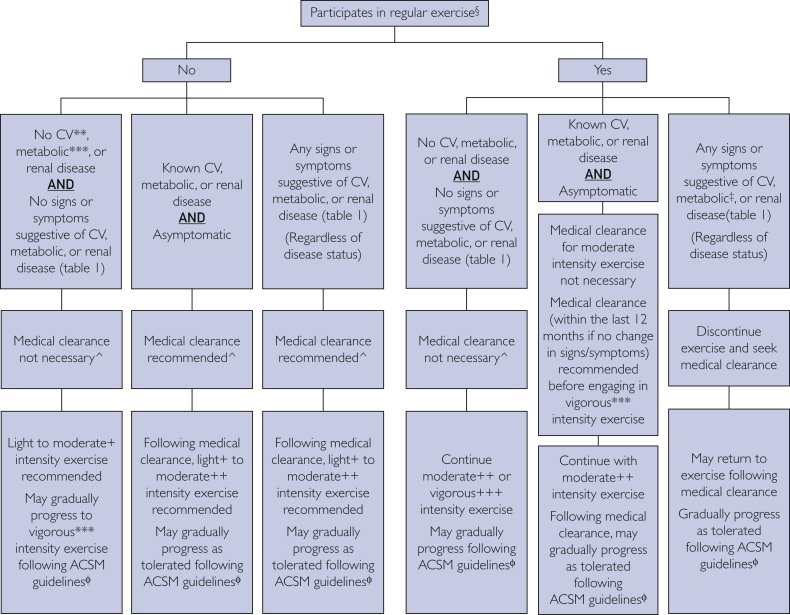
The ACSM exercise preparticipation health screening logic model. ^**§**^Exercise participation is defined as performing planned, structured physical activity at least 30 minutes at moderate intensity on at least 3 days per week for at least the last 3 months. ∗∗Cardiac, peripheral vascular, or cerebrovascular disease. ∗∗∗Type 1 or 2 diabetes mellitus. ˆMedical clearance is defined as approval from a health care professional to engage in exercise. +Light intensity exercise is defined as an intensity that causes slight increases in HR and breathing. ++Moderate intensity exercise is defined as an intensity that causes noticeable increases in HR and breathing. +++Vigorous intensity exercise is defined as an intensity that causes substantial increases in HR and breathing. ACSM = American College of Sports Medicine; CV = cardiovascular; HR = heart rate. ^ɸ^Adapted from *ACSM’s Guidelines for Exercise Testing and Prescription.*^15^^,^^16^

The purpose of the ACSM exercise preparticipation health screening is to identify individuals who may be at elevated risk for acute exercise-related sudden cardiac death and/or myocardial infarction during and for some time after exercising.^15^^,^^16^ The ACSM exercise preparticipation health screening is based upon the three primary factors that account for an acute exercise-related cardiovascular event that include the: (1) current level of physical activity; (2) presence of signs or symptoms suggestive of or having CVD or metabolic (ie, diabetes mellitus) or renal disease; and (3) desired exercise intensity. These three factors frame the ACSM exercise preparticipation health screening recommendations and determine if medical clearance is needed before exercise participation. The algorithm is depicted in Figure 1 and overviewed below. Furthermore, we developed an easy-to-use checklist for determining the presence of signs and symptoms suggestive of CVD or metabolic or renal disease (see Table 1).

**Table 1:** Major Signs or Symptoms Suggestive of Cardiovascular, Metabolic, and Renal Disease^a^^b^^c^^d^

Signs/symptoms suggestive of disease	Image	Description of the signs/symptoms suggestive of disease	Yes	No
Dizziness Difficulty breathing while laying down		Dizziness may be a sign of disease when in combination with stumbling or difficulty walking, fainting, a blackout, numbness or weakness, SOB, a sudden or severe headache, chest pain or an irregular heart rate, a very stiff neck, vomiting, or seizures; or if dizziness is recurrent and prolonged. Difficulty breathing lying down (orthopnea) and is relieved with sitting or standing.		
Forceful or fast heartbeat		Forceful or fast heartbeat (palpitations or tachycardia; heart rate >100 beats/min). Unpleasant awareness of a rapid heart rate at rest that may be associated with SOB, lightheadedness, rapid pulse rate, chest pain, or fainting.		
Ankle or limb pain or swelling		Swelling of the foot, ankle, and/or limb (ankle edema). May appears as puffiness, stretched or shiny skin, skin that retains a dimple (pits) after being pressed, or increased abdominal size.		
Chest related pain or discomfort Claudication/pain in legs		Pain/discomfort in the chest/neck/arm/other area that may result from an reduced oxygen supply to the heart (myocardial ischemia). Feeling of squeezing, constricting, burning, or “heaviness” behind/below/across the chest, in the front of the body; in one or both arms or the shoulders; in the neck, cheeks or teeth; or in the forearms or fingers, and/or interscapular region of the back. Provoked by exertion, excitement, other forms of stress, the cold weather, or after meals. Pain in the legs that is due to an inadequate blood supply (intermittent claudication). Symptoms are brought on by exercise and do not occur with standing or sitting. Pain often described as a burning or cramp which disappears within 1-2 minutes after stopping exercise.		
Shortness of breath		SOB at rest or with mild exertion; and/or unusual fatigue during regular daily activities is abnormal if occurs at a level of exertion not expected to evoke symptoms.		
Murmur or heart sound		Murmur may be a sign of cardiovascular disease when combined with blue skin especially in the fingertips and lips, swelling and sudden weight grain, SOB, enlarged neck veins, chest pain, dizziness, or fainting.		

Adapted from ACSM’s Guidelines for Exercise Testing and Prescription (pp 26-27).^16^

a SOB = shortness of breath.

b These signs or symptoms must be interpreted within the clinical context in which they occur because they are not all specific for cardiovascular, metabolic, or renal diseases.

c If the answer is yes to any sign or symptom medical clearance is required.

d May be used in combination with the Questionnaire for Exercise Professionals from *ACSM’s Guidelines for Exercise Testing and Prescription* (p 36).^16^

Regularly physically active asymptomatic patients without known CVD or metabolic or renal disease may continue their exercise program without medical clearance and progress gradually as tolerated according to the ACSM FITT ExR_x_ guidelines. Physically active asymptomatic patients with known CVD or metabolic or renal disease whose health care provider has cleared them to exercise within the last year do not need to consult with their health care provider to continue with a moderate-intensity exercise program unless they develop resting or exertional symptoms suggestive of CVD or metabolic or renal disease, or experience a change in health status. Physically active patients who develop signs or symptoms suggestive of CVD or metabolic or renal disease should discontinue exercise and obtain medical clearance before resuming their exercise program.

Physically inactive but otherwise healthy asymptomatic patients may begin a light-to-moderate-intensity exercise program without medical clearance, and in the absence of symptoms, can progress gradually as recommended by the ACSM FITT ExR_x_ guidelines. Physically inactive patients with known CVD or metabolic or renal disease, and/or those with signs or symptoms suggestive of these diseases, should obtain medical clearance before starting an exercise program.

### Step 2. Identify the CVD Risk Factors

A significant departure in the current ACSM exercise preparticipation health screening recommendations from past recommendations was removal of CVD risk factors. Reasons for doing so was that their predictive value for an acute exercise-related cardiac event was low, and including them resulted in excessive referrals to a health care provider to obtain clearance before exercise participation, which is a deterrent to adopting and maintaining an exercise program.^21^^,^^22^ Nonetheless, the ACSM scientific roundtable expert members acknowledged identifying CVD risk factors should remain an important part of designing the FITT ExR_x_ for disease prevention and management.^15^^,^^23^^,^^24^ Table 2 contains the CVD risk factors and their defining criteria.^2^, ^3^, ^4^, ^5^, ^6^, ^7^, ^8^ To perform step 2, the presence or absence of the CVD risk factor is denoted, and the number of CVD risk factors is totaled (see Table 2).

**Table 2:** The Cardiovascular Disease Risk Factors and Defining Criteria^a^^,^^b^

Risk factors	Defining criteria	Yes or No	AHA CVH score
Age, years	Men ≥45; women ≥55		
Family history	Myocardial infarction, coronary revascularization, or sudden death before 55 years old in father or other male first-degree relative or before 65 years old in mother or other female first-degree relative.		
Cigarette smoking	Current cigarette smoker or those who quit within the previous 6 months or exposure to environmental tobacco smoke.		
Physical inactivity	Not participating in at least 30 minutes of moderate intensity physical activity on at least 3 days of the week for at least 3 months.		
Obesity	BMI ≥30 kg/m^2^ or waist girth >102 cm (40 in) for men and >88 cm (35 in) for women.		
Hypertension	Systolic ≥130 mm Hg and/or diastolic ≥80 mm Hg BP, confirmed by measurements on at least two separate occasions, or on antihypertensive medication.		
Dyslipidemia	LDL-C ≥130 mg/dL (3.37 mmol/L) or HDL-C <40 mg/dL (1.04 mmol/L) or on lipid-lowering medication. If total serum cholesterol is all that is available, use ≥200 mg/dL (5.18 mmol/L).		
Diabetes	Fasting BG ≥126 mg/dL (7.0 mmol/L) or 2-hour plasma glucose values in oral glucose tolerance test ≥200 mg/dL (11.1 mmol/L) or HbA1C ≥6.5%.		
Negative risk factor HDL-C ≥60 mg/dL (1.55 mmol/L)^c^			
Total number of cardiovascular disease risk factors			

Adapted from ACSM’s Guidelines for Exercise Testing and Prescription (p 48).^16^

a AHA = American Heart Association; BG = blood glucose; BMI = body mass index; BP = blood pressure; CVH = cardiovascular health; HbA1C = hemoglobin A1C; HDL-C = high-density lipoprotein cholesterol; LDL-C = low-density lipoprotein cholesterol.

b If the presence or absence of a cardiovascular disease risk factor is not disclosed or is not available, that cardiovascular disease risk factor should be counted as a risk factor.

c High HDL-C is considered a negative risk factor. For individuals having high HDL-C ≥60 mg/dL (1.55 mmol/L), for these individuals one positive risk factor is subtracted from the sum of positive risk factors.

### Step 3. Prioritize the CVD Risk Factor to Design the FITT ExR_x_

**Figure 2:**
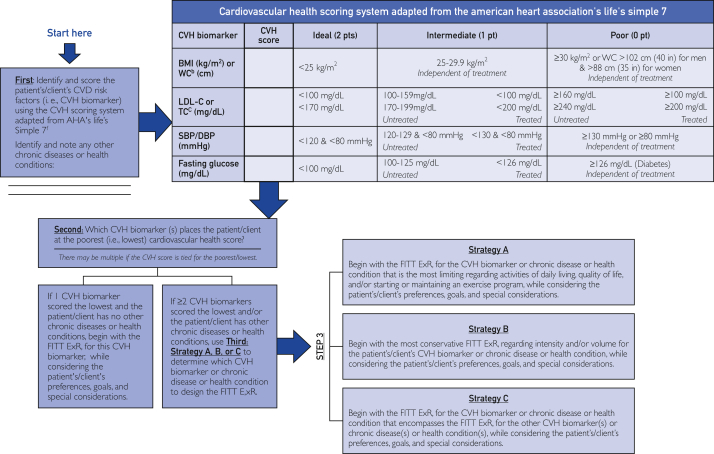
A clinical decision support system for prioritizing the cardiovascular risk factor or chronic disease or health condition to design the FITT ExR_x_. ^a^AHA = American Heart Association; BMI = body mass index; CVH = cardiovascular health; CVD = cardiovascular disease; DBP = diastolic blood pressure; ExR_x_ = exercise prescription; FITT = frequency, intensity, time, and type; SBP = systolic blood pressure; WC = waist circumference. ^b^CVH score cut-offs for WC are based on *ACSM’s Guidelines for Exercise Testing and Prescription* (p 70).^16^^c^Total cholesterol is only scored if low-density lipoprotein–cholesterol (LDL-C) is not available. ^d^The strategy chosen should be based on the CVD risk factor and/or chronic disease and health condition with the FITT ExR_x_ that best fits that strategy’s description, while considering the patient’s preferences, goals, and special considerations (Table 3). ^e^There is a strong, inverse association between AHA Life's Simple 7 ideal metrics and the incidence of myocardial infarction, stroke, coronary heart disease, and other cardiovascular outcomes, as well as noncardiovascular conditions such as depression, cognitive function, and cancer.^2^^f^The CVH scoring system has been adapted from its original version^18^^,^^19^ to accommodate: (1) adjustments in scoring for resting values that are being treated by medication, as any CVD risk factor that is being treated by medication is regarded as having that CVD risk factor independent of the resting value; and (2) the 2017 AHA/American College of Cardiology blood pressure guidelines.^8^^g^See Table 3 and the *ACSM’s Guidelines for Exercise Testing and Prescription*^16^ (pp 268-376) for the FITT ExR_x_ and special considerations for each CVD risk factor and/or chronic disease and health condition.

Prioritizing the CVD risk factor consists of two parts as shown in Figure 2 and is described below.

#### Step 3a

*Score the CVD Risk Factors With the AHA*_*7*_*CVH.* Using the AHA_7_CVH in Figure 2, the CVD risk factors identified in step 2 are scored in Table 2 as ideal (2 points), intermediate (1 point), or poor (0 points) CVH if the patient is untreated or being treated with medication for the major CVD risk factors of obesity (ie, BMI, and if not available, waist circumference), dyslipidemia (ie, low-density lipoprotein–cholesterol [LDL-C], and if not available, total cholesterol), hypertension (ie, BP), and diabetes mellitus (ie, fasting BG).

If the patient has only one of these CVD risk factors, and there are no other chronic diseases or health conditions to consider, the ExR_x_ is designed for that CVD risk factor using the ACSM FITT principle of ExR_x_ as described in step 4.^16^ If the patient presents with two or more CVD risk factors and only one emerges with the poorest (ie, lowest) AHA_7_CVH score, and there are no other chronic diseases or health conditions to consider, the ExR_x_ is designed for that CVD risk factor using the ACSM FITT principle of ExR_x_ as described in step 4.^16^ If the patient presents with two or more CVD risk factors and two or more emerge with the same poorest AHA_7_CVH score, and/or the patient has other chronic diseases or health conditions to consider, the ACSM strategies for designing an ExR_x_ for people with multiple CVD risk factors who may have other chronic diseases and health conditions are then used to determine which CVD risk factor or chronic disease or health condition to focus the FITT ExR_x_.^20^

#### Step 3b

*Apply the ACSM Strategies.* The adapted ACSM strategies for designing an ExR_x_ for people with multiple CVD risk factors and/or chronic diseases and health conditions are shown in Figure 2 and described below.^20^ It is possible that more than one strategy may be used to prioritize the CVD risk factor to design the FITT ExR_x_. In that case, the purpose of P3-EX is to guide the clinician in deciding which CVD risk factor to focus the FITT ExR_x_. However, P3-EX is not meant to replace good clinical judgement on which strategy is best to use. Furthermore, to maximize safety and therapeutic benefit, the FITT ExR_x_ should be delivered in collaboration with a properly credentialed exercise professional with a focus on transitioning to ongoing self-managed exercise.

##### ACSM Strategy A

Begin with the CVD risk factor tied for the poorest AHA_7_CVH score that is the most limiting regarding performing activities of daily living, quality of life, and/or starting or maintaining an exercise program, while considering the patient’s preferences, goals, and special considerations.^20^ An example for which strategy A would be used to prioritize the CVD risk factor to design the FITT ExR_x_ is a patient who has diabetes-related neuropathy with a fasting BG of 136 mg/dL and LDL-C of 162 mg/dL, both of which receive a score of 0 on the AHA_7_CVH. For this patient, the FITT ExR_x_ for diabetes mellitus would be recommended as outlined in step 4 based upon strategy A because of the limitations imposed by diabetes-related neuropathy in performing activities of daily living.

In some cases, other chronic diseases and health conditions could take precedent over CVD risk factors to design the FITT ExR_x_ based on strategy A. For example, for a patient with severe osteoarthritis regardless of the number of CVD risk factors and AHA_7_CVH scores, the FITT ExR_x_ for osteoarthritis would be recommended based on strategy A because of the limitations imposed by severe osteoarthritis in performing activities of daily living.

##### ACSM Strategy B

Begin with the CVD risk factor tied for the poorest score on the AHA_7_CVH whose FITT ExR_x_ is the most conservative in terms of exercise intensity, volume and/or total time, while considering the patient’s preferences, goals, and special considerations.^20^ An example for which strategy B would be used to prioritize the CVD risk factor for the FITT ExR_x_ is a patient who is physically inactive and has a BMI of 32 kg/m^2^ and BG of 128 mg/dL confirmed on two occasions, both of which receive a score of 0 on the AHA_7_CVH. For this patient, the FITT ExR_x_ for diabetes mellitus would be recommended as outlined in step 4 based on strategy B because the FITT ExR_x_ for diabetes mellitus is more conservative in terms of exercise intensity, volume, and total time than is the FITT ExR_x_ for obesity.^16^

In some cases, other chronic diseases and health conditions could take precedent over CVD risk factors to design the FITT ExR_x_ based on strategy B. For example, for a patient with fibromyalgia, regardless of the number of CVD risk factors and AHA_7_CVH scores, the FITT ExR_x_ for fibromyalgia would be recommended based on strategy B because of the very conservative nature of the FITT ExR_x_ for fibromyalgia in terms of exercise intensity, volume, and total time.

##### ACSM Strategy C

Begin with the CVD risk factor tied for the poorest AHA_7_CVH score whose FITT ExR_x_ encompasses the FITT ExR_x_ for the other CVD risk factor(s) in terms of exercise intensity, volume, and/or total time while considering the patient’s other chronic diseases or health conditions, preferences, goals, and special considerations.^20^ An example for which strategy C would be used to prioritize the CVD risk factor for the FITT ExR_x_ is a patient who has a BMI of 31 kg/m^2^ and BP of 136/88 mm Hg, both of which receive a score of 0 on the AHA_7_CVH. For this patient, the FITT ExR_x_ for obesity would be recommended as outlined in step 4 based on strategy C because the FITT ExR_x_ for obesity encompasses the FITT ExR_x_ for HTN in terms of exercise intensity, volume, and time.^16^ In addition, obesity is a major CVD risk factor for HTN and targeting obesity would favorably impact HTN.^25^

In some cases, other chronic diseases and health conditions could take precedent over the CVD risk factors to design the FITT ExR_x_ based on strategy C. For example, for a healthy older adult who has multiple CVD risk factors regardless of their score on the AHA_7_CVH, the FITT ExR_x_ for healthy older adults could be recommended because this FITT ExR_x_ encompasses the FITT ExR_x_ for any CVD risk factors that this older adult may encounter.

### Step 4. Design the FITT ExR_x_

Once the CVD risk factor or chronic disease or health condition has been prioritized with the AHA_7_CVH^18^^,^^19^ and ACSM strategies in Figure 2, the ACSM FITT ExR_x_ for that prioritized CVD risk factor and/or chronic disease or health condition is then recommended.^16^ The ACSM FITT ExR_x_ for the major CVD risk factors of diabetes mellitus, dyslipidemia, hypertension, and obesity is detailed in Table 3. The reader is referred to other resources for additional information on the FITT ExR_x_ for these CVD risk factors and other chronic diseases and health conditions.^16^^,^^26^

**Table 3:** The ACSM FITT ExR_x_ for Diabetes Mellitus, Dyslipidemia, Hypertension, and Obesity^a^

Diabetes mellitus^b^				
	Aerobic	Resistance	Flexibility	Neuromotor
Frequency, days/week	3-7	A minimum of 2 nonconsecutive days/week, but preferably 3.	≥2-3	≥2-3
Intensity	Moderate (40%–59% VO_2_R or 11-12 RPE rating) to vigorous (60%–89% VO_2_R or 14-17 RPE rating).	Moderate (50%-69%% of 1-RM) to vigorous (70%-85% of 1-RM).	Stretch to the point of tightness or slight discomfort.	Not determined.
Time	T1DM: 150 min/wk at moderate intensity, or 75 min/wk at vigorous intensity, or combination. T2DM: 150 min/wk at moderate to vigorous intensity.	At least 8 to 10 exercises with 1-3 sets of 10-15 repetitions to near fatigue per set early in training. Gradually progress to heavier weights using 1-3 sets of 8-10 repetitions.	Hold static stretch for 10-30 s; 2-4 repetitions of each exercise.	≥20-30 min/d.

Dyslipidemia^c^				
Frequency, days/week	≥5 to maximize caloric expenditure.	2-3	≥2-3	≥2-3
Intensity	40%-75% VO_2_R or HRR.	Moderate (50%-69% of 1-RM) to vigorous (70%-85% of 1-RM) to improve strength; <50% 1RM to improve muscle endurance.	Stretch to the point of tightness or slight discomfort.	Not determined.
Time	30–60 min/d. To promote or maintain weight loss, 50-60 min/d or more of daily exercise is recommended.	2-4 sets, 8-12 repetitions for strength; ≤ 2 sets, 12-20 repetitions for muscular endurance.	Hold static stretch for 10-30 s; 2-4 repetitions of each exercise.	≥20-30 min/d.

Hypertension^d^				
Frequency	2-3 d/wk	2-3 d/wk	≥2-3 d/wk	≥2-3 d/wk
Intensity	Moderate intensity, (i.e., 40% - 59% VO_2_R or HRR; RPE 12-13 (on a 6–20 scale) to Vigorous (i.e., 60% - 80% VO_2_R or HRR; RPE 14-16 (on a 6–20 scale).	60% - 70% 1-RM; may progress to 80% 1-RM. For older individuals and novice exercisers begin with 40-50% 1RM.	Stretch to the point of feeling tightness or slight discomfort.	Low to Moderate
Time	≥ 30 min/d of continuous or accumulated exercise. If intermittent exercise performed, begin with a minimum of 10 min bouts.	2-4 sets of 8-12 repetitions for each of the major muscle groups.	Hold static stretch for 10-30 s; 2-4 repetitions of each exercise.	≥20-30 min/d

Obesity^e^				
Frequency, days/week	≥ 5	2-3	≥2-3	≥2-3
Intensity	Initial intensity should be moderate (40%-59% VO_2_R or HRR); Progress to vigorous (≥60% % VO_2_R or HRR) for greater health benefits.	60%-70% of 1 RM; Gradually increase to enhance strength and muscle mass.	Stretch to the point of feeling tightness or slight discomfort.	Not determined.
Time	30 min/d (150 min/wk); Increase to 60 min/d or more (250-300 min/wk).	2-4 sets of 8-12 repetitions for each of the major muscle groups.	Hold static stretch for 10-30 s; 2-4 repetitions of each exercise.	≥20-30 min/d

a ACSM = American College of Sports Medicine; FITT = frequency, intensity, time, and type; ExR_x_ = exercise prescription; HRR = heart rate reserve; 1-RM = one repetition maximum; RPE = rating of perceived exertion; T1DM = type 1 diabetes mellitus; T2DM = type 2 diabetes mellitus; VO_2_R = oxygen uptake reserve.

b Adapted from *ACSM’s Guidelines for Exercise Testing and Prescription* (p 271).^16^

c Adapted from *ACSM’s Guidelines for Exercise Testing and Prescription* (p 278).^16^

d Adapted from *ACSM’s Guidelines for Exercise Testing and Prescription* (p 281).^16^

e Adapted from *ACSM’s Guidelines for Exercise Testing and Prescription* (p 289).^16^

Please see the Supplemental Material (available online at http://mcpiqojournal.org) that shows how P3-EX is applied to a case study of a patient with multiple CVD risk factors. After applying the ACSM FITT ExR_x_ for the prioritized major CVD risk factor or chronic disease or health conditions, the patient’s special considerations including medication use and side effects are compiled and the FITT Ex R_x_ is adjusted. A description of the special considerations for the major CVD risk factors and/or chronic diseases and health conditions to adjust the FITT ExR_x_ is beyond the scope of this report. The reader is referred to other resources for detailed information on special considerations in ExR_x_ for these major CVD risk factors.^16^^,^^26^

## Conclusion

With the release of the *Physical Activity Guidelines for Americans, 2*^*nd*^ *Edition*,^27^ there is now a call to action for physicians and health care providers to recommend physical activity to their patients because physical activity is the “best buy” for our health.^13^ The US Department of Health and Human Services recommends clinicians provide patients with regular counseling on physical activity and promote physical activity as one of the singularly most effective preventive health interventions available.^28^ Yet, to the best of our knowledge, a clinical decision support system for clinicians to use to prescribe exercise to their patients does not exist.^11^, ^12^, ^13^ To address this critical unmet need we have developed an evidenced-based, guided, and time-efficient tool for clinicians to use to prescribe exercise for patients with multiple CVD risk factors who may have other chronic diseases and health conditions founded upon industry standard recommendations of the AHA and ACSM (ie, P3-EX). Future directions include continued testing of P3-EX within a university-based online graduate program^29^ as well as further investigation to better establish its feasibility, acceptability, and clinical utility as an ExR_x_ tool.
