# Supplementary material for: Development of a Novel Clinical Decision Support System for Exercise Prescription Among Patients With Multiple Cardiovascular Disease Risk Factors
Source: Mayo Clin Proc Innov Qual Outcomes. 2020 Oct 22;5(1):193–203. doi: 10.1016/j.mayocpiqo.2020.08.005 (PMC7930885; doi:10.1016/j.mayocpiqo.2020.08.005)
Supplement: Supplemental Material [file mmc1.pdf]

Supplemental Appendix 1. The Exercise Prescription Clinical Decision Support System (*P3-EX*) Applied to a Case Study

We now illustrate how clinicians can apply *P3-EX* to a case study of a patient with multiple CVD risk factors.

Jackson is a 50 year old corporate executive who is 5 feet 10 inches and weighs 220 pounds (BMI 31.6 $kg•m^{-2}$). He would like to join the fitness center at work to improve his health by restarting his walking program which he abandoned 6 months ago. His last physical examination had the following results: total cholesterol 240 $mg•dL^{-1}$, LDL-C 134 $mg•dL^{-1}$, HDL-C 44 $mg•dL^{-1}$, fasting BG 130 $mg•dL^{-1}$ confirmed from his physical examination last year, BP 128/84 mmHg, and waist circumference 38 inches. His mother recently had a bypass at 75 years. Jackson is a nonsmoker and had been a regular walker, but for the last 6 months when he could only find the time to walk 1 day on the weekend for 30 minutes. At his last physical examination, over a year ago, his physician expressed concern to Jackson about his strong family history of Type 2 diabetes mellitus and emphasized the importance of regular exercise to avoid going on medication. Since that physical examination Jackson has put on 10 pounds which has motivated him to start walking regularly again.

Step 1. Complete the ACSM Exercise Preparticipation Health Screening

The questions to consider in the ACSM exercise preparticipation health screening process to determine if Jackson needs medical clearance are detailed in Figure 1 and include: (1) Does Jackson participate in regular exercise? *(No)*; (2) What is Jackson's preferred exercise intensity? *(Moderate)*; and (3) Does Jackson have any signs or symptoms of (Table 1) *(No)* or the presence of CVD or metabolic or renal disease? *(Yes)*. The answers to these questions indicate that Jackson requires medical clearance before starting his walking program at work due to being physically

inactive for the past 6 months and having metabolic disease or diabetes mellitus with a BG of 130 mg•dL^{-1} confirmed on at least two or more occasions.

Step 2. Identify the CVD Risk Factors

Supplemental Table 1 lists the CVD risk factors and their defining criteria as they apply to Jackson. In denoting the presence or absence of each CVD risk factor by comparing Jackson's biometric values to the defining criteria, he has six CVD risk factors that include age, physical inactivity, obesity, hypertension, dyslipidemia, and diabetes mellitus.

Step 3. Prioritize the CVD Risk Factor to Design the FITT ExR$_x$

Step 3a. Score the CVD Risk Factors with the AHA$_7$CVH

Begin by scoring Jackson's CVD risk factors according to the AHA$_7$CVH in Figure 2. Jackson's AHA$_7$CVH scores are entered into Supplement Table 1 showing that BMI (i.e, obesity) and diabetes mellitus (i.e., BG) received the same lowest score of *0*, while BP (i.e., hypertension) and LDL-C (i.e., dyslipidemia) received the same score of *1*.

Step 3b. Apply the ACSM Strategies

Obesity and diabetes mellitus received the same lowest score so that the ACSM strategies for individuals with multiple CVD risk factors and/or chronic diseases and health conditions in Figure 2 are now applied. Because Jackson's CVD risk factors are not limiting him, using Strategy A to focus the FITT ExR$_x$ is not applicable to him. Moving onto Strategy B, when comparing the FITT ExR$_x$ for diabetes mellitus and obesity (Table 3), the FITT ExR$_x$ for diabetes mellitus is more conservative in terms of exercise intensity and volume than obesity. Therefore, using Strategy B in Figure 2, the FITT ExR$_x$ for diabetes mellitus for someone who is physically inactive would be most appropriate for Jackson. It is possible that an alternative ACSM strategy could be used to prioritize the CVD risk factor so that Strategy C could be considered as well for Jackson. The

FITT ExR_x for obesity does encompass the FITT ExR_x for diabetes mellitus. However, choosing Strategy B because the FITT ExR_x for diabetes mellitus is more conservative than obesity in terms of exercise intensity and volume would be the most appropriate FITT ExR_x for Jackson at this time since he is physically inactive.

Step 4. Design the Targeted FITT ExR_x

The prioritized CVD risk factor that emerged from applying the AHA_7CVH and ACSM strategies for multiple CVD risk factors in Figure 2 to Jackson is diabetes mellitus. The corresponding FITT ExR_x for diabetes mellitus in Table 3 is then prescribed for Jackson. The reader is referred to other resources for detailed descriptions of the FITT ExR_x for diabetes mellitus and associated special considerations.(4,26)

Supplemental Table 1. The Cardiovascular Disease Risk Factors and Defining Criteria Applied to Jackson

**Risk Factors[a]**	**Defining Criteria**<br>**Jackson's Biometric Values**	**Yes or No**	**AHA CVH Score**
**Age**	Men ≥45 yr; women ≥55 yr<br>**Jackson is 50 yr**	Yes	NA
**Family history**	Myocardial infarction, coronary revascularization, or sudden death before 55 yr in father or other male first-degree relative or before 65 yr in mother or other female first-degree relative<br>**Jackson's mother had coronary revascularization at 73 yr**	No	NA
**Cigarette smoking**	Current cigarette smoker or those who quit within the previous 6 mo or exposure to environmental tobacco smoke<br>**Jackson is a nonsmoker**	No	NA
**Physical Inactivity**	Not participating in at least 30 min of moderate intensity physical activity on at least 3 d of the week for at least 3 mo<br>**Jackson walks one d per week for 45 min**	Yes	NA
**Obesity**	BMI ≥30 $kg \cdot m^{-2}$ or waist girth >102 cm (40 in) for men and >88 cm (35 in) for women<br>**Jackson's BMI is 31.6 $kg \cdot m^{-2}$**	Yes	0
**Hypertension**	Systolic ≥130 mm Hg and/or diastolic ≥80 mm Hg BP, confirmed by measurements on at least two separate occasions, or on antihypertensive medication	Yes	1

	**Jackson's BP is 128/84 mmHg**		
**Dyslipidemia**	LDL-C ≥130 mg•dL^{-1} (3.37 mmol • L^{-1}) or HDL-C [a] <40 mg•dL^{-1} (1.04 mmol•L^{-1}) or on lipid-lowering medication. If total serum cholesterol is all that is available, use ≥200 mg•dL^{-1} (5.18 mmol•L^{-1})<br>**Jackson has a LDL-C of 134 mg•dL^{-1}**	Yes	1
**Diabetes**	Fasting BG ≥126 mg • dL^{-1} (7.0 mmol•L^{-1}) or 2 h plasma glucose values in oral glucose tolerance test ≥200 mg•dL^{-1} (11.1 mmol•L^{-1}) or HbA1C ≥6.5%<br>**Jackson fasting BG is 130 mg•dL^{-1}**	Yes	0
**Negative Risk Factor** HDL-C ≥60 mg•dL^{-1} (1.55 mmol•L^{-1})[c]<br>**Jackson's HDL-C is 44 mg•dL^{-1}**		No	**NA**
**Jackson's Total Number of Cardiovascular Disease Risk Factors**		6	**NA**

[a]AHA= American Heart Association; BG=blood glucose; BMI=body mass index; BP= blood pressure; CVH=cardiovascular health; HDL-C=high-density lipoprotein cholesterol; LDL-C=Low-density lipoprotein cholesterol; NA=not applicable; P3-EX= the prioritize personalize prescribe exercise clinical decision support system

[b]If the presence or absence of a cardiovascular disease risk factor is not disclosed or is not available, that cardiovascular disease risk factor should be counted as a risk factor

[c]High HDL-C is considered a negative risk factor. For individuals having high HDL-C ≥60 mg•dL^{-1} (1.55 mmol•L^{-1}), for these individuals one positive risk factor is subtracted from the sum of positive risk factors

[d]Adapted from *ACSM's Guidelines for Exercise Testing and Prescription*[(16)] p. 48

[e]This supplemental appendix has not been edited, and the authors take responsibility for the accuracy of all data.
